# What really is nontokenistic fully inclusive patient and public involvement/engagement in research?

**DOI:** 10.1111/hex.14012

**Published:** 2024-03-15

**Authors:** Andrea Hilton, Molly Megson, Aidin Aryankhesal, Jessica Blake, George Rook, Anne Irvine, Jinpil Um, Anne Killett, Ian Maidment, Yoon Loke, Jayden van Horik, Chris Fox, TIMES programme team

**Affiliations:** ^1^ Faculty of Health Sciences, School of Paramedical Peri‐Operative and Advanced Practice University of Hull Hull UK; ^2^ Academy of Primary Care, Hull York Medical School University of Hull Hull UK; ^3^ Faculty of Medicine and Health Sciences, School of Health Sciences University of East Anglia Norwich UK; ^4^ TIMES Research Exeter UK; ^5^ University of Exeter Medical School Exeter UK; ^6^ Aston Pharmacy School, College of Health and Life Sciences Aston University Birmingham UK

**Keywords:** epistemic justice, inclusivity, patient and public involvement and engagement

## Abstract

Patient and public involvement and engagement (PPIE) is critically important in healthcare research. A useful starting point for researchers to understand the scope of PPIE is to review the definition from the National Institute for Health and Care Research (NIHR) as, ‘research being carried out “with” or “by” members of the public rather than “to”, “about” or “for” them’. PPIE does not refer to participation in research, but to actively shaping its direction. The ‘Effectiveness of a decision support tool to optimise community‐based tailored management of sleep for people living with dementia or mild cognitive impairment (TIMES)’ study is funded through the NIHR programme grant for applied research. TIMES has thoroughly embraced PPIE by ensuring the person's voice is heard, understood, and valued. This editorial showcases how the TIMES project maximised inclusivity, and we share our experiences and top tips for other researchers. We base our reflections on the six key UK standards for public involvement; Inclusive Opportunities, Working Together, Support and Learning, Communications, Impact and Governance. We present our work, which had been co‐led by our PPIE leads, academics and partners including, together in dementia everyday, Innovations in Dementia, The UK Network of Dementia Voices (Dementia Engagement & Empowerment Project) and Liverpool Chinese Wellbeing. We have a Lived Experience Advisory Forum on Sleep, which includes people with dementia, family carers, representatives of the South Asian Community and the Chinese community.

## 1 INTRODUCTION

The significance of the patient and user voice in health research has seen patient and public involvement and engagement (PPIE) methods graduate from a methodological consideration to a requirement for many funding bodies. Research in which PPIE is embedded is an active partnership between patients, family and nonfamily carers, members of the public, and researchers; with the overarching aim of positively influencing and shaping the research plan and its delivery.^1^ PPIE is a continuous and reflexive process that must be planned and embedded before research delivery begins, through to dissemination and impact. A useful starting point for researchers to understand the scope of PPIE is to review the definition given by the National Institute for Health and Care Research (NIHR) as, ‘research being carried out “with” or “by” members of the public rather than “to,” “about” or “for” them’.^2^ Theories of epistemic justice^3^ recognise the need for a person's voice to be both heard (testimonial) and understood in the interpretation of meaning (hermeneutic).

The ‘Effectiveness of a decision support tool to optimise community‐based tailored management of sleep for people living with dementia or mild cognitive impairment’ (TIMES)^4^ is an NIHR programme grant for applied research. TIMES has thoroughly embraced PPIE by ensuring the person's voice is heard, understood and valued at every stage. This editorial showcases how the TIMES project ensured inclusivity, and we share our experiences and top tips for future researchers.

This editorial is structured around the Standards for Public Involvement in Research from the NIHR (United Kingdom)^5^—Inclusive Opportunities, Working Together, Support and Learning, Communications, Impact, Governance.

### 1.1 Standard—Inclusive opportunities

For TIMES, recruitment for PPIE members began by working closely with members of patient representative organisations including together in dementia everyday (tide), and Innovations in Dementia/UK Network of Dementia Voices (Dementia Engagement & Empowerment Project). People living with dementia (PLwD) or mild cognitive impairment (MCI) were involved from the beginning, before funding, in the initial study design. Recruitment via established organisations had the benefit of ensuring our potential PPIE members were interested and already familiar with the research. However, we also recognised the potential for a marginalised perspective and exclusion of other narratives in limiting recruitment to those already engaged in research. Therefore, we widened our inclusion through snowball sampling to recruit new members as our meetings continued, and members of our groups shared their experiences with members of their personal social circles. We sought the advice and support of our PPIE partners here such as together in dementia everyday and Innovations in Dementia. We also broadened our recruitment by promoting the study through the NIHR's People in Research, with a specific focus on attracting South Asian PPIE members for a more diverse representation in the study.

Throughout our ongoing development, we have continued to promote diversity in our groups and sought assistance with our partner dementia organisations to recruit representatively. We also have dedicated separate PPIE groups in recognition and respect of cultural nuances and the diverse needs of our population. We actively seek out and incorporate diverse narratives, ensuring that the richness and complexity of the experiences of PLwD are accurately reflected in the research.

As a research team, we remain aware of the potential of unconscious biases that could obscure our interpretative lens. We continue to learn and reframe our understanding of dementia through our PPIE group, such as the difficulty of the Chinese community accessing primary care. We equally encourage the development of confidence and identity in the PPIE group members as active and valued equals in our research project. This is evident in the impact standard with our PPIE presenting at the conference.

*Our top tip*: Approach engagement and recruitment to PPIE as an ongoing, open format to widen and diversify inclusion opportunities.

### 1.2 Standard—Working together

Our PPIE comprised separate Lived Experience Advisory Forums (LEAFs) for people living with dementia and people with caring experience, as well as a separate South Asian and Chinese community. Groups were facilitated and conducted using a cooperative structure, not led by the research team. This promoted equal participation opportunities between members not impacted by potential power dynamics which might have been perceived by researcher‐led activities. Conducting groups separately, as proposed by PLwD and carers, allowed the PPIE process to focus on relevant issues raised according to experience and need.

Sessions began with an introduction to the TIMES study and current updates: our aims and anticipated outcomes of the research and suggested methodologies. For the Chinese group, there were language barriers, necessitating the delivery of sessions through an interpreter, and all materials were pretranslated. This language challenge, coupled with cultural preferences, led to a preference for sharing experiences within their community rather than with outsiders. However, during the course of TIMES, it was clear that online meetings were challenging if the meetings were conducted through an interpreter. Therefore, we supplemented the online meetings with face‐to‐face where members of the research team travelled to the group. During these early meetings, we also discussed the role of PPIE in the TIMES project to define and record the purpose and expectations of the groups. Early meetings allowed PPIE groups to establish roles and responsibilities, identifying a group lead from the PPIE cohort to chair future meetings.

We explored potential ways of working together and established online meetings which enabled a wide selection of members across the United Kindom; indeed our PPIE co‐chairs stated without travel people are comfortable in their own homes, also travel can be a burden. The research team initially proposed quarterly meetings, which were amended to monthly as the PPIE groups felt that more frequent, ‘bite‐size’ sessions were preferable. We recognised the challenges with online meetings, which were emphasised as a particular issue for PLwD/MCI, and as such we made sure to structure regular breaks during our sessions and allow extra time for other discussions and getting to know one another. We also recognised that members of our PPIE groups often navigate busy personal schedules and as such meetings were also conducted on a drop‐in basis, informally and formally to suit individual availability, thus enabling maximum inclusivity, with the depth of involvement varying. This was all in response to suggestions made by our PPIE leads—see support and learning.

*Our top tip*: Encourage a member‐led, co‐operative structure of management. Researchers are involved as equal contributors or indeed as learners.

### 1.3 Standard—Support and learning

‘Support and Learning’ are crucial for ensuring meaningful engagement. We prioritised knowledge assimilation, especially for those new to dementia research such as what does involvement look like. Moreover, with our team's international composition, we focussed on understanding language nuances and cultural values. Learning through PPIE is a mutual experience, as we learnt to reframe our understanding of dementia to respect individual differences and the impact a diagnosis can have. PPIE also gave our team opportunities to learn about the research processes from the perspective of the public who were involved which promoted shared understandings of the role of PPIE in dementia research.

*Our top tip*: Simplify communication (send gentle reminders) and tailor information provision and data collection. We used premeetings before the actual meetings so people know what to expect; these premeetings were preparatory, giving PPIE members a chance to ensure they had an understanding of agenda items scheduled for the main meeting, and provided the opportunity to ask basic questions in a ‘safe’ environment. These pre‐meetings also have the added benefit of enabling those carers who are unable to attend the formal meeting itself to make a contribution. Drop‐in sessions were introduced at the request of carers to keep in touch with the research and up to date on developments in between the formal meeting. It is also important not to leave it too long into the study to reconnect, keeping people involved along the way.

### 1.4 Standard—Impact

Throughout TIMES, our PPIE groups have made active decisions which have informed and driven the research in relation to methodology and intervention co‐design. As part of our dissemination of findings, we invited PPIE group co‐chairs to engage with conference events in person and via video testimonials to share the personal impact of their PPIE experiences. This provided authentic accounts to share with the research community and encouraged the development of individual identities as valued members of the research project. Engagement with academic impact therefore acknowledges potential power relations and transfers decision making in outputs.

The main benefits of our PPIE approach have been to focus on the practicalities of carrying out research on people with dementia. PPIE members brought their lived experience to bear on such matters as memory difficulties, length of questionnaires, selection of appropriate outcome measures, and understanding the many potential causes of sleep difficulties.

PPIE group co‐chairs recorded short films about their involvement which were presented in a session at the British Society of Gerontology annual conference in 2023. For further information/access please email a.hilton@hull.ac.uk directly.

*Our top tip*: Involve PPIE members in authorship of outputs and dissemination events and indeed ‘hand over’ authorship to PPIE members. It is essential to establish a PPIE group early in the setting up of a project, so that they can shape the research proposal and method from the start; often this is done too late to make changes that PPIE members might propose.

### 1.5 Standard—Communications

TIMES facilitated an open two‐way communication stream between the research team and PPIE members. TIMES ran monthly online drop‐in sessions in addition to the more formal quarterly LEAFs meetings, where experiences were shared, and key elements of the project were discussed. In addition to information sharing, these meetings were an opportunity for rapport building and peer support. Members identified priority areas to address within the study, which informed the initial formulation of the TIMES intervention and the selection of outcome measures for evaluation. Using the learning from these meetings, TIMES ran a series of workshops to co‐produce a decision support tool. PPIE members were first invited to attend a workshop designed to enable them to share their topic‐specific expertise in a familiar space. Members were then given the option to attend an online collaborative meeting with all stakeholders, which comprised PPIE members, healthcare professionals, and researchers.

Acknowledging a moral obligation, our team is committed to unwavering support for PPIE members. To keep PLwD/MCI informed and engaged, we transparently communicated the development of results at each stage. We have learnt to adjust our communication style to reduce jargon, acronyms and information overload. For example, we have reduced the number of slides (if a presentation was used) and offered flexibility in participation and diversified approaches (group video calls, email, phone calls, 1–1 video chats). Data collection tools were tailored with the unique needs of PLwD and MCI in mind, and sessions were deliberately kept short, not exceeding 1 h for focussed and effective engagement. Inclusivity was promoted by appointing interpreters for non‐English speakers, translating slides for the Chinese group into Cantonese. In recognition of the contributions of PLwD or MCI and their carers, our research team ensured fair reimbursement for their time and expertise, following approved financial models.

PPIE members were updated quarterly via a TIMES newsletter which we uploaded to the study website and also sent via email. Newsletters were presented in an easy‐read format and without jargon and importantly were co‐produced and reviewed by PPIE representatives. The newsletters aimed to engage and involve PPIE members who were unable or did not want to participate in online meetings.

TIMES recognised that some people lacked the confidence to attend online meetings. This was an issue identified by the Chinese PPIE group who due to translation and technology issues were therefore approached face‐to‐face to introduce the project. The meeting facilitators (Liverpool Chinese Wellbeing staff) also engaged in briefing and debriefing sessions with the group so that they could have some understanding of the materials before the meeting. Through debriefing sessions, participants provided feedback or additional comments they could not provide during the meeting. Additionally, the facilitator visited PPIE members’ homes to help them join the online meeting if they had not got access or invited them to their centre to join as a group. While it was not sustainable to have every meeting face‐to‐face, the group were then able to attend online meetings in their local meeting place and, as mentioned earlier, this was supplemented by face‐to‐face meetings.

*Our top tip*: Consider the reciprocal benefits of being a PPIE member—enable opportunities for rapport building and peer support. Be flexible and offer a range of communication methods. Remember that PPIE members get personal satisfaction and validation from their participation, but that they may need arrangements to be changed to meet their needs. This often simplifies working for all members and is welcomed by all.

### 1.6 Standard—Governance

Effort was taken throughout to ensure PPIE voices were heard, valued and respected in all decision making. We were reflective about potential unconscious bias. Within TIMES we have a structured governance pathway with reporting mechanisms. GR (author and TIMES co‐applicant as PPIE co‐lead) is an active member of the programme management group as well as co‐chairing the LEAFs groups as highlighted in the *working together*.

Our PPIE members are always involved in a pre‐meeting briefing session ensuring our documents or items for discussion are reviewed. We have all learnt from this; presentations have been changed and topics for discussion modified as a direct result of the voice of the PPIE members.

**Figure 1:**
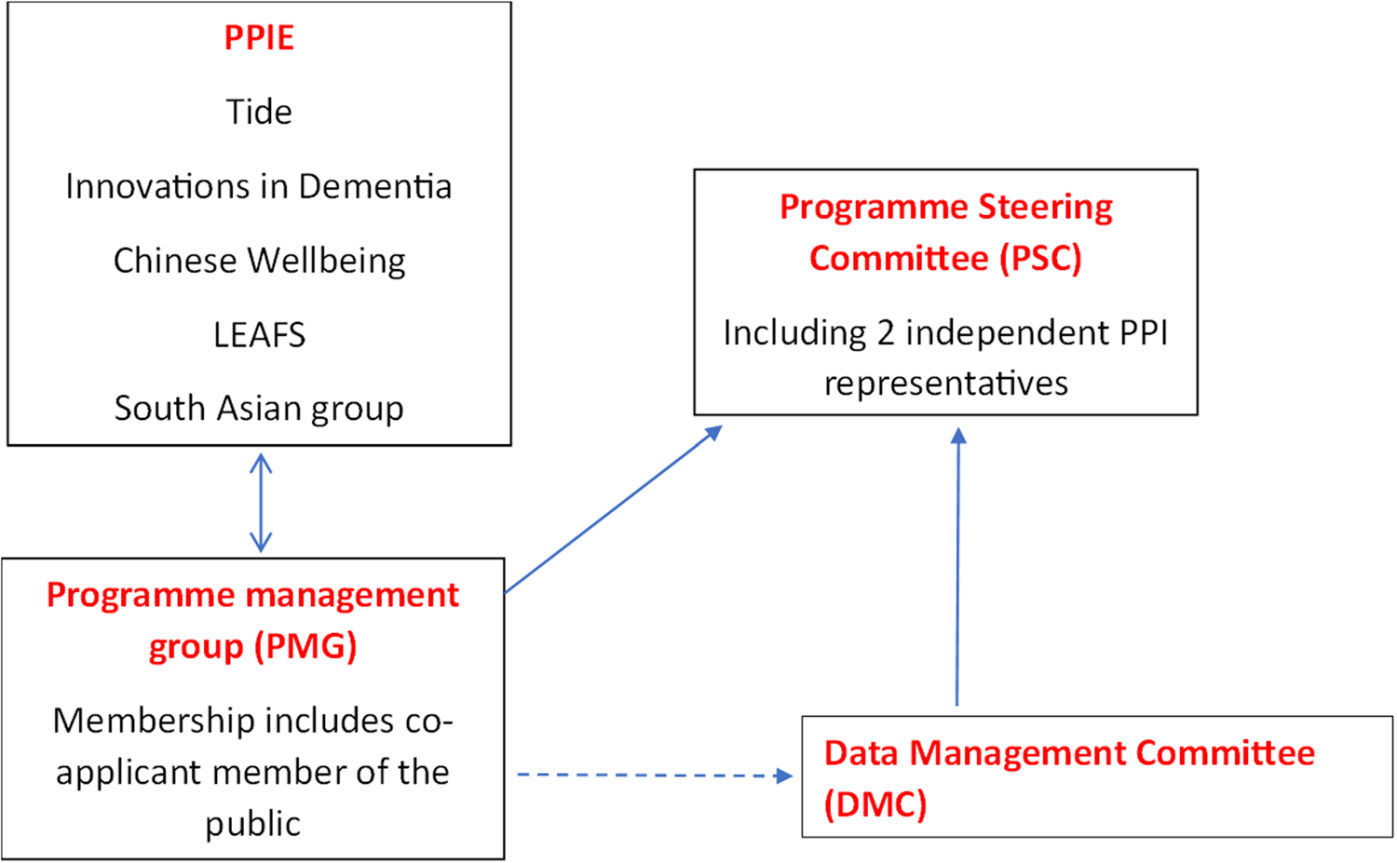
Simplified governance structure. LEAFs, Lived Experience Advisory Forums; PPIE, Patient and Public Involvement/Engagement.

There is a formal governance process and resources allocated for PPIE within TIMES with a budget which has supported the extensive PPIE work. The reporting governance structure below clearly demonstrates this standard (Figure 1).

*Our top tip*: Resources, particularly time and financial, cannot be underestimated. We have found with TIMES this is a major consideration. PPIE members must be supported to feel confident to voice concerns and be actively listened to. Significant time must be invested by the researchers to ensure this happens.

## 2 CONCLUSION

Our experiences with TIMES indeed reflect that nontokenistic, fully inclusive patient and public involvement/engagement in research with PLwD/MCI is possible. However, researchers should not underestimate the time and other resources involved including training and support that may be needed, funding in grant applications to reflect this cost and a research co‐applicant/researcher should be costed in to fully support a PPIE co‐applicant(s) in true partnership.

## AUTHOR CONTRIBUTIONS

Andrea Hilton, Molly Megson, Aidin Aryankhesal and Jessica Blake wrote the editorial, and all other authors critically reviewed and edited for content. Andrea Hilton, Molly Megson, Aidin Aryankhesal and Jessica Blake supported, contributed and were academically involved in PPIE for the TIMES study. George Rook and Anne Irvine were co‐PPI leads of the LEAFs groups, George Rook is a PPI co‐applicant. Jinpil Um, Ian Maidment and Yoon Loke were instrumental in supporting and facilitating the South Asian and Chinese PPIE groups. Andrea Hilton and Anne Killett supported the LEAFs groups with premeetings and during meetings, liaising with George Rook and Anne Irvine. Jayden van Horik centrally managed the trial and co‐ordinated all the extensive PPIE activity. Chris Fox is the chief investigator and facilitated all PPIE from inception. All authors approve the final version of this editorial.

## CONFLICT OF INTEREST STATEMENT

The authors declare no conflict of interest.

## Data Availability

Data are available from the corresponding author upon reasonable request.
